# Online Outreach Services Among Men Who Use the Internet to Seek Sex With Other Men (MISM) in Ontario, Canada: An Online Survey

**DOI:** 10.2196/jmir.4503

**Published:** 2015-12-09

**Authors:** David J Brennan, Nathan J Lachowsky, Georgi Georgievski, Brian R Simon Rosser, Duncan MacLachlan, James Murray, Cruising Counts Research Team

**Affiliations:** ^1^ University of Toronto Factor-Inwentash Faculty of Social Work University of Toronto Toronto, ON Canada; ^2^ University of British Columbia Faculty of Medicine Vancouver, BC Canada; ^3^ British Columbia Centre for Excellence in HIV/AIDS Vancouver, BC Canada; ^4^ University of Minnesota School of Public Health Minneapolis, MN United States; ^5^ Ontario HIV Treatment Network Toronto, ON Canada; ^6^ Ontario Ministry of Health and Long Term Care AIDS Bureau Toronto, ON Canada

**Keywords:** gay men, HIV prevention, Internet, online outreach, men who have sex with men, HIV/AIDS, sexual health, mobile technology and sexual health

## Abstract

**Background:**

Men who use the Internet to seek sex with other men (MISM) are increasingly using the Internet to find sexual health information and to seek sexual partners, with some research suggesting HIV transmission is associated with sexual partnering online. Aiming to “meet men where they are at,” some AIDS service organizations (ASOs) deliver online outreach services via sociosexual Internet sites and mobile apps.

**Objective:**

To investigate MISM's experiences and self-perceived impacts of online outreach.

**Methods:**

From December 2013 to January 2014, MISM aged 16 years or older were recruited from Internet sites, mobile apps, and ASOs across Ontario to complete a 15-minute anonymous online questionnaire regarding their experience of online outreach. Demographic factors associated with encountering online outreach were assessed using backward-stepwise multivariable logistic regression (*P*<.05 was considered significant).

**Results:**

Of 1830 MISM who completed the survey, 8.25% (151/1830) reported direct experience with online outreach services. Encountering online outreach was more likely for Aboriginal versus white MISM, MISM from Toronto compared with MISM from either Eastern or Southwestern Ontario, and MISM receiving any social assistance. MISM who experienced online outreach felt the service provider was friendly (130/141, 92.2%), easy to understand (122/140, 87.1%), helpful (115/139, 82.7%), prompt (107/143, 74.8%), and knowledgeable (92/134, 68.7%); half reported they received a useful referral (49/98, 50%). Few MISM felt the interaction was annoying (13/141, 9.2%) or confusing (18/142, 12.7%). As a result of their last online outreach encounter, MISM reported the following: better understanding of (88/147, 59.9%) and comfort with (75/147, 51.0%) their level of sexual risk; increased knowledge (71/147, 48.3%); and feeling less anxious (51/147, 34.7%), better connected (46/147, 31.3%), and more empowered (40/147, 27.2%). Behaviorally, they reported using condoms more frequently (48/147, 32.7%) and effectively (35/147, 23.8%); getting tested for HIV (43/125, 34.4%) or STIs (42/147, 28.6%); asking for their partners’ HIV statuses (37/147, 25.2%); and serosorting (26/147, 17.7%). Few MISM reported no changes (15/147, 10.2%) and most would use these services again (98/117, 83.8%). Most MISM who did not use online outreach said they did not need these services (1074/1559, 68.89%) or were unaware of them (496/1559, 31.82%).

**Conclusions:**

This is the first online outreach evaluation study of MISM in Canada. Online outreach services are a relatively new and underdeveloped area of intervention, but are a promising health promotion strategy to provide service referrals and engage diverse groups of MISM in sexual health education.

## Introduction

### Background

Globally, the HIV epidemic continues to disproportionately burden sexual minority men. In Canada, this includes men who identify as gay, bisexual, two-spirit (used by the Aboriginal community to describe sexual minority individuals and/or nonbinary gender identity), as well as other men who have sex with men who may not necessarily identify as gay or bisexual—hereafter, all sexual minority men will be referred to as GB2M. In 2011 in Canada, there was an estimated 71,300 people living with HIV and nearly half of those were GB2M (n=33,330); nearly half of new HIV infections in 2011 were among GB2M (1480/3175, 46.61%) [1]. These figures have not changed much since 2008 [1]. As well, nearly one in five of all HIV-positive GB2M are unaware of their HIV infection [1]. Ontario—Canada’s most populated province—accounts for the largest proportion (40.9%) of all HIV-positive tests reported nationally [2]. In Ontario, 60% of all people living with HIV/AIDS (PHA) are GB2M, who also accounted for 73% of all new diagnoses among men in 2012 [3].

### Sexual Minority Men, HIV, and the Context of the Internet

GB2M have been using the Internet and online mobile technologies for well over 20 years to connect with one another for social and sexual relations [4]. Because of advancements in these technologies, men who use the Internet to seek sex with other men (MISM) have adapted to evolving technology that provides new options for connecting and obtaining sexual health information [4]. Even though many jurisdictions have advanced human rights for lesbian, gay, and bisexual people, many GB2M live, work, and socialize in contexts where same sex activity is stigmatized, and sexuality and sexual behavior are restricted or met with disapproval [5]; thus, many resort to online and mobile technologies to communicate and interact with other GB2M [6]. In a 2013 telephone interview study, 49.9% of GB2M in Canada reported using the Internet to look for sex in the past 6 months and 14% used a sociosexual mobile app (eg, Grindr) [7]. In another Canadian study published in 2013 but conducted online, 88% of participants used the Internet to find sex partners [8]. These two figures likely provide the bounds of the true estimate. Regardless, the Internet is the most frequently used resource for sex (eg, one-third of Ontario GB2M surveyed in 2006 used the Internet at least once a week to look for sex) [9].

In particular, these technologies offer what Cooper has referred to as the *Triple A Engine* effect [10]. That is, using online and mobile technologies for communication with other MISM for social and sexual reasons is appealing and common because of three factors: Affordability, Accessibility, and Anonymity. Subsequent additions to this model include Acceptability (greater tolerance online) [11], Approximation (greater ability to experiment) [12], and most recently Affirmation (explore and confirm one’s identity) and Assessment (ability to prescreen and assess compatibility of potential partners) [13]. The components of this descriptive framework can assist researchers and providers in understanding how online outreach may be an important and beneficial tool to address online HIV/sexually transmitted infection (STI) prevention and sexual health promotion among MISM.

### Online Partners and Sexual Behavior

GB2M use electronic media to look for sex, friendship, and connection online. GB2M who seek sexual partners online report high rates of behaviors associated with HIV risk (eg, unprotected anal intercourse [UAI] with a serodiscordant partner) [14]. Among a 2008 community-based sample of GB2M in British Columbia, Canada, men who sought partners online were more likely to report 10 or more sexual partners in the past year compared with those who did not seek partners online [15].

### Online HIV/Sexually Transmitted Infection Prevention

Some AIDS service organizations (ASOs), community-based organizations (CBOs), public health groups, and Internet providers have developed programs and models that work directly with MISM to support sexual health. MISM appear to lack basic knowledge of HIV (eg, how HIV is transmitted and how condoms should be used), have had questions about HIV testing, and feel that community resources do not meet their needs [16]. Previous research has shown that the majority of MISM hold favorable attitudes toward online health promotion [14,17-19]. Online sexual health promotion can be an effective and low-cost method to educate MISM [18,20,21]. A 2013 study of MISM aged 18-24 years in Southern California found that the number one reason for using Grindr, a sociosexual mobile app, was to meet hookups (for sex); 70% of young MISM expressed a willingness to participate in app-based HIV prevention [22]. Online interventions have demonstrated a reduction in UAI [23], particularly among unknown or serodiscordant partners [24]. Other research has demonstrated efficacy in online interventions to increase HIV/AIDS knowledge, self-efficacy, and condom use among MISM [25]. As participants, MISM expect online health promotion to respect the online culture, build trust, and deliver well-crafted and focused messages [16]. A recent systematic literature review [26] on Internet and mobile app use for sexual health promotion among MISM in Canada highlighted the need for more research that examines this phenomenon and its implications. Specifically, though agencies are offering online outreach services, we know very little about how these online outreach services are understood, accessed, or relevant for the sexual health of MISM in Canada.

### Online Outreach to Men Who Use the Internet to Seek Sex With Other Men in Ontario, Canada

The ASO and sexual health sectors, as well as public health providers and public health scientists, have suggested a need to reconceptualize online media as vital tools for HIV prevention [4,27]. Though the Internet has been used in a number of ways for outreach to MISM (eg, e-blasts, social media, and chat rooms) over the past two decades, this study is focused on the notion of online outreach services that involve trained staff and/or volunteers logging into online sites and apps and responding to questions from, and providing referrals for services to, MISM in these environments. For the purposes of this research, online outreach is broadly defined as the delivery of information and/or support services regarding HIV, STIs, and general health with a particular focus on sexual health via any Internet website, mobile phone app, or Web-based tool that MISM use as a means of connecting with other MISM for social/sexual activity. Resources and geography limit comprehensive online outreach for MISM in Ontario. In terms of geographic coverage, while there is some Internet-based service provision in all regions of Ontario, 70% of online outreach is provided by agencies in Toronto, the province’s most populous metropolitan area [3]. It is important to note that for resource reasons, most of this online outreach is conducted at varying times of the day, including weekends, weekdays, and weeknights, depending on the provider's capacity and availability. It is not a 24-hour service and it is not available through all apps or websites. Some app or website providers prohibit this type of service on their sites as it interrupts the user experience. In Ontario, online outreach providers are generally either trained sexual health outreach workers or public health staff.

Online outreach has become a key tool in the delivery of sexual health information and services affecting MISM [3,8,22,28,29]. In light of the predominance of the Internet as a social and sexual venue for GB2M in Ontario, outreach in physical venues is no longer sufficient; online outreach can help reach more GB2M. This study developed out of consultations with community providers who were conducting online outreach to MISM. Often these providers were doing this outreach because they were aware that this is where the men they wished to reach were located, or because physical venues were not available or less popular. Given the numerous calls for more research directed at understanding how online tools can benefit sexual health promotion [26,30], few of these articles focused specifically on online outreach. Therefore, there is little evidence of the reach, depth, impact, or effectiveness of this type of outreach. Though ASOs and public health practitioners have developed apps specific to HIV prevention, these are infrequently downloaded and often poorly rated, suggesting that these apps may not be useful or that MISM prefer accessing information within the apps they use [31]. Agencies in Ontario have reported that “there is still some uncertainty about how to do Internet-based outreach well, as well as ongoing challenges in tracking and assessing the impact of this work” [3]. Several agencies report an increased demand for online outreach services [3]. Taken collectively, there is great interest in improving online outreach, but also some challenges conceptualizing, sustaining, and evaluating online media as effective learning environments.

Without better evidence and understanding of how MISM are seeking and/or experiencing online outreach, there remains a missed opportunity to connect with GB2M “where they are at” with the goals of preventing HIV/STI transmission and improving sexual health. This study sought to examine how MISM in Ontario access, experience, and perceive online outreach. That is, whether they find it useful, relevant, and applicable to their sexual health. The aim of this study was to assist in the development of tools that would be useful for sexual health online outreach for MISM.

## Methods

### Study and Participants

Data were drawn from a mixed-methods, community-based research study entitled *Cruising Counts*, which involved partnerships from across Ontario, Canada’s largest province. The research team included various frontline staff and managers from ASOs who were providing or had provided online outreach services to MISM, staff of the provincial Gay Men’s Sexual Health Alliance, a provincial HIV/AIDS health policy expert, and researchers from three academic institutions. A community advisory board composed of MISM met quarterly to inform and provide feedback on the research process, data collection tools, and knowledge translation activities. All study protocols were granted ethics clearance from the University of Toronto Research Ethics Board.

Between December 2013 and January 2014, participants were recruited from across Ontario using electronic advertisements on sociosexual websites (eg, Squirt.org, recon.com, bgclive.com, and craigslist.ca), mobile apps (eg, Grindr), social media (eg, Facebook and Twitter), and printed flyers distributed through ASOs. Participants were asked to complete an anonymous online questionnaire regarding their technology use, online behavior (sociosexual and health related), experience of online outreach, and demographics (see Table 1). To be eligible, participants must have sought sexual partners or sexual health information online in the past 6 months (or had an interest in doing so); identify as a gay, bisexual, two-spirit, straight, queer, or questioning cis- or transgender man; had in the past had sex with another man (or an interest in doing so); be at least 16 years old; and either lived or worked in Ontario or had visited Ontario at least three times in the past year. Participants were offered an opportunity to enter a random draw for one of 40 cash prizes of Can $100, which were delivered via Interac e-Transfer.

### Measures

#### Online Outreach

Our primary dependent variable was participants having experienced online outreach or not. Participants were prompted with the following definition: “By online outreach services, we mean that while you were online or using an app, you had any interactive conversations, whether in real time or not (can include chatting, responding to postings/message boards, or messaging), between you and an online outreach worker.” Participants were asked, “Have you ever encountered or used online outreach services?” and to indicate who initiated the contact (participant or provider). Those who were contacted by online outreach services were asked if they were told why they were being contacted, whether a photo was used (agency logo, personal, unsure), and whether they were comfortable being contacted.

All participants who had experienced online outreach rated several aspects of their last experience (see Table 2 for items) on a 5-point Likert scale from 1 (disagree) to 5 (agree), which were dichotomized into agree (4 or 5) or not (1-3). Participants were asked to indicate any changes that resulted from their last online outreach encounter (see Table 3 for items), if they would use online outreach services again (yes or no), and to explain why or why not through an open-ended text response. Those participants who had not encountered online outreach were asked to indicate why: *no need/interest*, *not available when needed*, *don’t trust*, or *don’t know about it or where to find it*. All participants were asked to provide qualitative feedback on any difficulties they had trying to access these online outreach services if these services were of interest to them.

#### Demographics

Independent variables for this analysis included age (in years), race/ethnicity (white, black, Latino, Aboriginal, South Asian, Southeast/East Asian, mixed race, or other), sexual orientation (gay/homosexual, bisexual, or other), gender (cis-gender male or not), two-spirit status (yes or no), trans person (yes or no), student (yes or no), social assistance (Ontario Disability Support Program [ODSP]/Employment Insurance [EI]/Ontario Works or not), highest level of formal education attained (no postsecondary education, some postsecondary education, or finished postsecondary education or any postgraduate education), immigration status (Canadian citizen/permanent resident or not), and self-reported HIV status (HIV negative, HIV positive, or unknown). Further, geographic location was grouped into major provincial regions (Toronto, Central Ontario, Southwestern Ontario, Eastern Ontario, and Northern Ontario) using participants' forward sortation areas (ie, first three characters of a Canadian postal code).

### Analyses

All quantitative data analyses were conducted using the statistical package Stata/SE version 13 (StataCorp) and *P<*.05 was considered significant unless otherwise specified. Data were analyzed to determine the prevalence of online outreach experience and associated factors. Descriptive statistics of the overall sample and for those participants who experienced online outreach were prepared. Independent factors associated with experience of online outreach were determined using logistic regression. Univariate analyses were conducted to screen independent variables using a liberal *P* value of .20 [32]. A final multivariate model was built using a manual backward-stepwise elimination approach [32]. Nonsignificant likelihood ratio tests were used to confirm removal of any categorical variables. Confounding was manually assessed throughout model building; if the addition or removal of a variable resulted in a greater than 30% change in any other independent variable’s coefficient, it was retained in the model [32]. A research assistant manually coded qualitative data, which were collected through open-ended text responses, with iterative consultations with the first and second author (DJB, NJL) and to resolve unclear coding questions.

## Results

Of the 1830 men who completed the online questionnaire, 151 men (8.25%) reported experience with online outreach, 95 men (5.19%) were unsure if they had, and 25 men (1.37%) refused to answer the question. Table 1 provides the descriptive statistics of the overall sample and the prevalence of, and univariate associations with, reporting online outreach experience. Two factors that were significant at the univariate level, but that were not included in the multivariable analysis, were HIV status and being two-spirited. Compared with HIV-negative men, HIV-positive men were more likely to have experienced online outreach (odds ratio [OR] 2.19, 95% CI 1.35-3.55). Also, two-spirit participants were more likely to have also experienced online outreach compared with those who were not two-spirit (OR 3.38, 95% CI 1.33-8.58). The final multivariate model included race/ethnicity, location, and social assistance as independent factors associated with having experienced online outreach (see right-most column in Table 1). Aboriginal men were more likely than white men to have experienced online outreach (adjusted odds ratio [AOR] 2.75, 95% CI 1.03-7.29). Compared with men who lived in Toronto, men in Southwestern Ontario (AOR 0.49, 95% CI 0.28-0.84) and Eastern Ontario (AOR 0.60, 95% CI 0.37-0.97) were less likely to have experienced online outreach. Finally, men who were receiving some form of social assistance (eg, disability or unemployment insurance) were more likely to have experienced online outreach than those who were not receiving social assistance (AOR 3.23, 95% CI 1.96-5.31).

**Table 1:** Sample demographics and the prevalence of, and factors associated with, online outreach experience.^a^

Demographics		Overall sample (n=1830), mean (SD) or n (%)	Experienced online outreach (n=151), mean (SD) or n (%)	Univariate associations, OR^b^ (95% CI)	Multivariate associations, AOR^c^ (95% CI)
Age in years, mean (SD)		37.8 (13.2)	36.6 (13.1)	0.99 (0.98-1.01)	Not included
**Race/ethnicity, n (%)**					
	White	1448 (79.13)	117 (78.5)	1.00	1.00
	Black	33 (1.80)	5 (3.4)	2.09 (0.79-5.52)	1.24 (0.41-3.73)
	Latino	45 (2.46)	4 (2.7)	1.13 (0.40-3.20)	0.95 (0.32-2.81)
	Aboriginal	27 (1.48)	6 (4.0)	3.38 (1.33-8.58)^d^	2.75 (1.03-7.29)^d^
	Other	29 (1.58)	2 (1.3)	0.90 (0.21-3.85)	0.87 (0.20-3.79)
	South Asian	40 (2.24)	2 (1.3)	0.59 (0.14-2.49)	0.58 (0.14-2.48)
	Southeast/East Asian	112 (6.12)	6 (4.0)	0.64 (0.28-1.50)	0.53 (0.21-1.36)
	Mixed race	70 (3.83)	7 (4.6)	1.27 (0.57-2.84)	1.14 (0.50-2.57)
**Sexual orientation, n (%)**					
	Gay	1325 (72.40)	117 (77.5)	1.00	Not selected
	Bisexual	438 (23.93)	33 (21.9)	0.85 (0.57-1.26)	
	Other	63 (3.44)	1 (0.7)	0.16 (0.02-1.20)	
**Two-spirit, n (%)**					
	No	1789 (97.76)	144 (95.4)	1.00	Not selected
	Yes	36 (1.97)	7 (4.6)	2.72 (1.17-6.32)^d^	
**Trans, n (%)**					
	No	1800 (98.36)	147 (97.4)	1.00	Not selected
	Yes	25 (1.37)	4 (2.7)	2.11 (0.72-6.24)	
**Cis-gender male, n (%)**					
	No	31 (0.05)	5 (3.3)	1.00	Not selected
	Yes	1795 (98.09)	146 (96.7)	0.47 (0.18-1.23)	
**HIV status, n (%)**					
	HIV positive	146 (7.98)	23 (15.4)	2.19 (1.35-3.55)^d^	Not selected
	HIV negative	1439 (78.63)	113 (75.8)	1.00	
	Unsure	217 (11.86)	13 (8.7)	0.74 (0.41-1.34)	
**Location, n (%)**					
	Toronto	512 (27.98)	55 (37.9)	1.00	1.00
	Central Ontario	315 (17.21)	24 (16.6)	0.69 (0.42-1.13)	0.67 (0.40-1.13)
	Southwestern Ontario	350 (19.13)	21 (14.8)	0.53 (0.31-0.89)^d^	0.49 (0.28-0.84)^d^
	Eastern Ontario	421 (23.00)	29 (20.0)	0.61 (0.38-0.98)^d^	0.60 (0.37-0.97)^d^
	Northern Ontario	121 (6.61)	16 (11.0)	1.27 (0.70-2.30)	1.03 (0.55-1.95)
**Education, n (%)**					
	High school or less	248 (13.55)	24 (15.9)	1.38 (0.83-2.29)	Not included
	Some postsecondary	855 (46.72)	74 (49.0)	1.18 (0.82-1.71)	
	Bachelor’s degree or greater	713 (38.96)	53 (35.1)	1.00	
**Student, n (%)**					
	No	1505 (82.24)	127 (85.2)	1.00	Not included
	Yes	295 (16.12)	22 (14.8)	0.87 (0.54-1.39)	
**Social assistance, n (%)**					
	No	1680 (91.80)	124 (83.2)	1.00	1.00
	Yes	120 (6.56)	25 (16.8)	3.37 (2.09-5.44)^d^	3.23 (1.96-5.31)^d^
**Canadian citizen/permanent resident, n (%)**					
	No	82 (4.48)	6 (4.0)	1.00	Not included
	Yes	1748 (95.52)	145 (96.0)	1.16 (0.50-2.72)	

^a^Missing values excluded from this table.

^b^OR: odds ratio.

^c^AOR: adjusted odds ratio.

^d^ *P*<.05.

Most men had contacted the service provider directly (125/151, 82.8%). For those who had been contacted by an online outreach worker (26/151, 17.2%), most participants reported that the worker explained why they were contacting them (16/26, 62%), that the worker had an agency logo (12/26, 46%) or photo of themselves (5/26, 19%), and that they were comfortable being contacted (17/26, 65%).

Participants rated their last online outreach experiences very positively (see Table 2). Men reported that the individual was friendly (130/141, 92.2%), used language they could understand (122/140, 87.1%), was helpful (115/139, 82.7%), was prompt to reply (107/143, 74.8%), and was knowledgeable and a trusted source of information (92/134, 68.7%). Over three-quarters of men were comfortable (116/144, 80.6%) and felt satisfied (110/142, 77.5%) with the interaction. Half of the men were provided with a useful referral (49/98, 50%). Very few men rated their last online outreach worker as confusing (18/142, 12.7%) or invasive or annoying (13/141, 9.2%).

**Table 2:** Participants’ ratings of their last online outreach encounter.^a^

Survey items	Agreed, n (%)
The individual was friendly (n=141)	130 (92.2)
The individual used language I could understand (n=140)	122 (87.1)
The individual was helpful (n=139)	115 (82.7)
I was comfortable with the interaction (n=144)	116 (80.6)
I was satisfied with the interaction (n=142)	110 (77.5)
The individual was prompt to reply (n=143)	107 (74.8)
The individual was knowledgeable and a trusted source of information (n=134)	92 (68.7)
The individual provided me with a useful referral (n=98)	49 (50)
The individual was confusing (n=142)	18 (12.7)
The individual was invasive or annoying (n=141)	13 (9.2)

^a^Missing values excluded from this table.

Table 3 shows the number and proportion of men who self-reported a variety of impacts as a result of their last online outreach experience. Over half of the men reported a better understanding of (88/147, 59.9%), and an increased comfort about (75/147, 51.0%), their level of sexual risk. Online outreach connected some men with a variety of referral services for men: 34.4% (43/125) got an HIV test, 28.6% (42/147) got an STI test, 12.2% (18/147) sought out counseling, 9% (2/22) of HIV-positive men sought HIV-related care, and 6.8% (10/147) of men got STI treatment. More frequent (48/147, 32.7%) and effective (35/147, 23.8%) use of condoms was also reported. Men also reported changes in their sexual partnering decisions with respect to HIV status: 25.2% (37/147) reported only having sex with partners whose HIV status they knew, 17.7% (26/147) reported only having sex with seroconcordant partners, and 4.8% (7/147) reported only having sex with HIV-positive partners whose viral load they knew. Online outreach also seemed to benefit men’s social, mental, and emotional well-being; some men felt less anxious (51/147, 34.7%) and others felt better connected (46/147, 31.3%), more empowered (40/147, 27.2%), and more sexually satisfied (20/147, 13.6%).

**Table 3:** Self-reported impact as a result of last online outreach encounter (n=147).^a^

Survey items		n (%)
I better understand my sexual risks		88 (59.9)
I am more comfortable about my level of sexual risks		75 (51.0)
I increased my knowledge		71 (48.3)
I feel less anxious		51 (34.7)
I got an HIV test (only for HIV-negative or status unknown men, n=125)		43 (34.4)
I use condoms more frequently		48 (32.7)
I feel better connected		46 (31.3)
I got an STI^b^ test		42 (28.6)
I feel more empowered		40 (27.2)
**I made decisions to**		
	...only have sex with people whose HIV status I knew	37 (25.2)
	...only have sex with people who had the same HIV status as I do	26 (17.7)
	...only have sex with HIV-positive people whose HIV viral load I knew	7 (4.8)
I use condoms more effectively (without slips, tears, or breakage)		35 (23.8)
I feel more sexually satisfied		20 (13.6)
I sought out counseling		18 (12.2)
I made no changes		15 (10.2)
I got HIV care (only for HIV-positive men, n=22)		2 (9)
I got STI treatment		10 (6.8)

^a^Missing values excluded from this table.

^b^STI: sexually transmitted infection.

The vast majority of participants (98/117, 83.8%; 34 refused to answer) who used online outreach services said that they would use them again. When asked to explain why qualitatively, 86 out of the 98 (88%) men provided reasons that were thematically coded; convenience (24/86, 28%), reliability (22/86, 26%), and anonymity (20/86, 23%) were the most commonly cited reasons for future use. For example, one man stated that these services were “available when needed (24 hrs) and voluntary,” while another explained that “the information was excellent and private.” Others reported they appreciated that these services offer “someone understanding, nonjudgmental, and open to discussing my concerns and answering my questions,” “immediate contact with a compassionate person,” and “human contact.” One man remarked supportively, “I find it difficult to ask the same questions with health care providers face-to-face because I have had negative, homophobic experiences in the past.”

The small minority of men who had previously used online outreach services, but indicated that they would not use them again (19/117, 16.2%), expressed three main reasons for this: (1) negative experiences or perceptions of these services, (2) long wait times to get a response, and (3) a preference or opportunity to interact with health professionals in person. One man stated that the experience “was invasive and [I] couldn’t feel as though I could trust them.” With regard to responsiveness, participants explained that they were “too slow to answer,” “received no response,” and that “the first time I tried contacting someone [in] real time they never replied back.” Finally, some of these men spoke positively about their current access to gay-friendly health services: “It was easier to just go to the nearest health clinic” and “I have an excellent support system with my doctor already.”

Overall, the men who accessed online outreach services rated the services as helpful and several explained how these services were becoming important sources of community and service information. For example, one man explained, “I find it difficult sometimes to remember when drop-in anonymous testing hours/locations happen, and the reminder that there is one in my 'hood is nice.” Participants provided a number of recommendations to consider in the future provision or adaptation of online outreach services. First, even among those who had accessed online outreach services, several men expressed that these services were not readily visible or available. One man stated that these services “need [a] more visible presence online” and another explained, “The only downside is that the individual has to seek out and discover the services available to them—so having this sort of thing more accessible on apps...would be a good idea.” Several men expressed an interest in more real-time conversations: “I wish the interaction could be simultaneous. My responses often had a long delay (like a day or two).” Others explained this in terms of better geographic, temporal, and online venue coverage: “They were helpful with answer[ing] my questions but...they could not refer me to local ones,” “more outreach volunteers in different locations,” and “They need to be on the sites that we are on, and to be there round the clock 24/7.”

Participants who had explicitly not experienced online outreach (n=1559) said they had not used these services because they had no need or interest (1074/1559, 68.89%), did not know about or where to find them (496/1559, 31.82%), did not trust them (85/1559, 5.45%), or the services were not available when they needed them (76/1559, 4.87%). The few respondents who said they did not trust these services explained that “I'm somewhat intimidated by the lack of privacy,” “Trust would be an issue, as well what personal information would be needed before the advice is given,” and they “need complete secrecy.” Many of the individuals who said that they did not need or were not interested in these services explained that “I go to the clinic about once a year and talk with nurses” or “I prefer to talk with a professional in person!”

All participants were asked to comment on any difficulties they had in trying to access online outreach services. Of 1830 men, 1005 (54.92%) were not interested in accessing these types of services; a further 412 (22.51%) had never experienced any barriers to accessing these services. Of those men who had not accessed online outreach and indicated that they wished to, but had potential difficulties, half of the reasons were either not knowing how to access these services (75/296, 25.3%) or being unaware that these services existed prior to this questionnaire (67/296, 22.6%). Participants reported that they “did not know they [online outreach services] existed or where to find them—I really never heard of this before, otherwise I would have contacted them” and “I had no idea these services existed...so more promotion of these services in the gay community would have been nice.”

Others indicated geographic difficulties with accessing these services. Several participants from smaller cities explained this poses almost paradoxical problems where gay-friendly services are not available (eg, “No access near where I live”), but attempts to make them available pose particular challenges to anonymity. One man explained that he lived in a “small city that hires mostly LGBT [lesbian, gay, bisexual, and transgender] staff. This will not help accessing services as who wants to run into a friend or acquaintance when seeking assistance?” Another participant stated, “In my small town, I still find that looking [for] and getting sexual health info or treatment has a huge stigma attached to it. Traveling to [Toronto] is costly and I can’t do it easily.” Access to Toronto-based services was also a challenge described for men comparably closer: “Services seem to be located downtown Toronto and I live in the suburbs.” Other geographic barriers were related to technology access and coverage: “limited Internet service where I live” and “Unless you’re located in the core of Toronto [major metropolitan center], it's likely you won’t see an outreach worker on Grindr.” Some participants expressed that they “don’t know how to tell if info is reliable,” are concerned with “having to give personal information...and feeling like you’re being judged,” or that the “gay outreach community is too small and not sure if what I share will remain confidential.”

## Discussion

### Principal Findings

This is the first study to examine the perceptions, expressed need, and self-reported impact of online outreach services for MISM (GB2M) in a Canadian context. Similar to findings in the United States [33], our study suggests that online outreach is a useful and important tool in HIV prevention for GB2M. The findings suggest that 8% of MISM in this sample have accessed online outreach services. The community members of the research team considered the reaching of 8% of all GB2M (MISM) through online outreach in their communities to be very successful, since the size of the population online is so large and since they represent a handful of agencies that have been offering these services only in the past couple of years. It is significant that participants are encountering these services in a space online where they are not necessarily going to seek health information and services. Most importantly, our analyses showed online services to be disproportionately used by GB2M who are hard to reach using other means, including HIV-positive men and Aboriginal men. Though several other studies focused primarily on youth [22,25], our findings suggest that there were no differences based on age. It would be useful to ensure broad age ranges for studies examining GB2M and online use.

In univariate analyses, two-spirit men and HIV-positive men were significantly more likely to access online outreach, and for trans-identified men this analysis approached significance. However, in multivariable analysis, Aboriginal participants (not necessarily those who identified as two-spirit) were more likely to access online outreach compared with white men. This may be a useful distinction for agencies serving Aboriginal men, because regardless of how they identify—gay, bisexual, two-spirit—this suggests Aboriginal men of all identities—gay, bisexual, two-spirit—are more likely to access resources available online. Like non-Aboriginal communities, stigma among Aboriginal populations regarding sexual minorities [34] may leave some two-spirit men feeling that online outreach is the preferred place to encounter the Triple A Engine effect of service usage because it is accessible, affordable, and anonymous. Those in Southwestern and Eastern Ontario were less likely than those in Toronto to access outreach. This may be a result of the numerous agencies providing online outreach in Toronto, whereas smaller communities may likely have only one (if any) agency providing such outreach. However, there were no differences in online outreach uptake between men in Northern Ontario, which has more rural and remote regions, and men in Toronto; this indicates the utility of these services to reach men who may be more geographically isolated [25] from both physical communities and in-person health and social services that are often clustered within large urban centers.

Those on social assistance were more likely to access online outreach than those who were not. This may speak to the affordability and accessibility of online resources and the lack of physical barriers/challenges to access them. These factors are important considerations for programming and policy implications when developing strategies in a variety of local jurisdictions and with specific populations. Indeed, a diverse array of singular and multiple outcomes were noted, including a decrease in anxiety, an increase in condom use, and even a better connection to community. These outcomes suggest online outreach has the capacity to address a comprehensive and integrated approach to care and services. Clearly, the needs go beyond specific condom use issues to those involving access to testing, pre- and post-HIV exposure treatment, and issues related to mental health and social well-being. There is a growing consensus that to address sexual health among GB2M, providers must understand the linkages between HIV/STIs, other health issues, and the social determinants of health along with the notion that multiple health epidemics—substance use, childhood trauma and bullying, mental health—are part of a fabric of syndemics that impact the health and wellness of GB2M across the life spectrum. These findings suggest the use of the Internet and other mobile technologies is a yet unrealized potential tool to link GB2M to rapidly evolving HIV-prevention information, such as the meaning of an undetectable viral load in relation to HIV transmission, and care, such as pre- and postexposure treatment. In addition, these online tools can also be formulated to address the underlying psychosocial factors impacting syndemics among GB2M [27]. Our findings support previous calls to encourage a more holistic approach to the health of MISM [4,27].

Of the men who accessed online outreach, their experiences were mostly favorable, finding the contact helpful and relevant, and reporting that they would use the service again. Half reported that they increased their knowledge on sexual health. Many men reported that they received a referral for testing or other services as an outcome. Nearly 90% of men who reported connection with online outreach reported some change as a result of that interaction. A recent systematic review of the literature has shown that using online tools for HIV prevention among gay and bisexual men were very effective at creating behavior change related to HIV transmission among gay and bisexual men [35].

Of the small number who would not use online outreach again, they primarily reported that they had a negative experience, a long wait time, or a preference for in-person contact. For those who did not access online outreach services, they reported mostly that they did not have a need or interest in the service, they did not know the service existed, or the services were not available at the time required. This feedback is useful when considering the development and implementation of online outreach services. Some regions may be lacking in online outreach due to resource constraints. In addition, MISM may be on certain apps or sites that have little or no online outreach and thus may not be coming into contact with such services. In our sample, most men reported being on numerous sites/apps (data not shown). Of course, it is not feasible that online outreach services can be provided on all sites or apps at all times, but, similar to previous research [28], it appears that MISM are interested in these services and a greater saturation of services may be helpful. It may be beneficial to have a sense of where men are appearing online and attempt to target the most popular sites/apps for a particular population or location, although these also shift over time as new products are released on the market. Therefore, it is important that agencies be aware of these changes in their population and are able to transition/adapt service provision across various platforms, including new and emerging ones [31].

Some men preferred the anonymity of the Internet for health care resources while others felt they had a trusting relationship with their providers. Thus, having resources online and on apps may be an important adjuvant to in-person care [28]. These services cannot and should not be a replacement for in-person care, but it seems clear there is a high demand and interest in online outreach, and that these services are commonly used by diverse MISM who may experience barriers to traditional services [8].

A large proportion of men reported not accessing these services due to a lack of need or interest (1074/1559, 68.89%). Future research would do well to understand the ways in which these men feel they have their health needs met to see if their resources, skills, or knowledge are transferable to other men. They may be using other forms of outreach, have advanced health literacy, or have access to other pertinent health information and care. It may also be that these men may have heightened HIV/STI risk, but do not perceive themselves to be at risk.

### Limitations

These findings were primarily self-reported and therefore may be impacted by recall and response bias. Participants were recruited through online venues and agency email blasts and therefore may not accurately capture the characteristics or number of GB2M who are online or using online outreach services. Our findings may not be generalizable to MISM populations across Ontario or in other jurisdictions, but they do provide an indication of important trends that should be investigated in population-based studies and with different study designs. In addition, the small sample size of men who accessed services may mask some of the other differences. Though there were location, race, and socioeconomic differences in those accessing online outreach, our study design could not help us to understand what the reason for these differences were. Finally, the scope of our conclusions is also limited by the lack of information related to funding history, reasons for starting and ending online outreach programs, and issues of program sustainability.

### Comparisons With Prior Work

Similar to previous research [36], our findings suggest that MISM are actively using the Internet for information regarding HIV and other STIs. Given that previous research has suggested that GB2M in online environments may lack basic HIV education [17], this study's findings resonate with previous research that suggests that GB2M are increasingly using the Internet and mobile technologies for sexual contact and sexual health promotion and information. Our findings also show that GB2M are indeed open and willing to use a variety of online resources to access HIV/STI and other health information [4,33]. Previous research has reported this acceptability in a variety of contexts, specifically online forums [18], Web tools [24], mobile-based apps [22], social media networks (ie, Facebook and YouTube) [37], and highly interactive online virtual environments [23]. The authors attempted to compare the levels of interaction and acceptability of online outreach to other forms of outreach among other GB2M. Though there is robust research focused on the outcomes and use of other forms of outreach, such as bar and bathhouse testing and counseling, there is a paucity of recent research that evaluates the acceptability levels of these tools among GB2M, thus limiting any possibility to compare. However, evidence suggests that previously developed mobile apps aimed at reducing HIV and other STIs have not been rated very well and were not frequently downloaded [31]. It is possible that instead of developing separate apps, engagement in online outreach within currently used apps may be more acceptable to MISM. Future research is needed to examine this possibility. Future research is also needed to test the efficacy and effectiveness of online outreach as a health intervention using experimental or quasi-experimental designs. An economic analysis of online outreach as a health service could help demonstrate the breadth of potential positive outcomes that occur. Additional useful research would be to examine the ways in which those who did not need or desire online outreach understand their sexual health and what they do to maintain it. Comparative research aimed at testing the levels of acceptability to various forms of online outreach for GB2M would provide beneficial data to ascertain the relevance and need for online versus more traditional forms of outreach (eg, bars and bathhouses).

Previous research has also suggested that rural GB2M are willing and interested in online tools for HIV/STI prevention and that such interventions can be efficacious as well [25]. Given Ontario’s large rural areas, it would be important for future research to examine the particular needs and usefulness of online outreach for rural and nonmetropolitan MISM. Our findings suggest that some GB2M desire to have resources accessible in person. This finding resonates with Hottes et al [31] who found that online testing was unlikely to replace in-person HIV testing among gay men, but may be a useful option for some who lack access to resources that are knowledgeable about gay men’s sexual health.

From a theoretical perspective, we found that Cooper’s Triple A Engine effect [10] and its offshoot components [11-13] were especially relevant to the experience of MISM using online outreach services. The elements of Cooper’s work and others who have developed it further [10-13] generally correspond with the data found in our study. These include that online outreach services are affordable, accessible (if one has a mobile phone or Internet connection), and anonymous. Though participation in the study required Internet access, any outreach experienced would be anonymous and free (affordable). In terms of accessibility of the services themselves for those online, the main barrier was a lack of awareness of the service or how to find it. This can help providers to consider better ways to increase awareness of their services. Other components related to the Triple A Engine effect suggested by other authors include Acceptability, Affirmation, and Assessment. Acceptability [11] was moderately evident in this study and future research should more directly examine whether having online outreach in apps is acceptable to GB2M; other research has shown that GB2M are interested in using online tools and resources for sexual health [22,28,31]. Affirmation [13] of oneself and a connection to a community were reported by those who felt they could trust the community providers. Assessment [13] of one’s sexual risk and making changes to one’s behavior as a result of this outreach was reported by most of the men who had encountered online outreach. Clearly, the use of online outreach represents an important and emerging tool to support the sexual health of MISM.

### Conclusions

Online outreach for HIV/STI prevention is a promising tool for GB2M and the agencies that serve them. For GB2M who access them, these services are helpful, provide useful referrals, and appear to provide some self-reported change in knowledge and behavior. For those who did not use online outreach, the primary reason was that they felt it was not necessary. For some men the response time was too slow or the services were challenging to find. These responses suggest providers might be interested in ensuring shorter response times and more awareness campaigns that inform GB2M about the scope and availability of their online and Web-based services. Shorter response times are important as GB2M who may be exposed to HIV may want to access postexposure prophylaxis (PEP) treatment, which should begin as soon as possible (within 72 hours) of exposure.

These findings suggest that online outreach services show great promise to reach some hard-to-reach populations. Further research may be needed to understand why men from certain regions, or those who are Aboriginal or on social assistance, may use online outreach services more. It is possible that those with marginalized status may have fewer local resources available to them and therefore using the Internet for information and referrals is more convenient and accessible. As per the expanded Triple A Engine effect concepts, because online outreach services are typically free and anonymous, this may be driving the increased usage among those with lesser financial resources and those who are closeted or more likely to suffer stigma. These findings also suggest opportunities for funders and service providers of online outreach to increase the reach and the uptake of such services to reduce the impact and burden of HIV and other STIs among GB2M. Additionally, because various mobile apps and websites cater to specific populations, online outreach has the advantage of providing more population-specific and individualized responses. The outreach can be provided in such a way as to specifically address one person’s current needs within an understood culture. Examples of this include Aboriginal men, men of color, rural men, younger men, and older men.

Finally, these findings suggest that future HIV-prevention interventions aimed at GB2M consider the further development and coverage of online outreach programs and services. Given the positive appreciation of this type of outreach and the accessibility and anonymity it provides, online outreach is an important and emerging tool that has the potential to address the broad range of issues that fuel the HIV epidemic among GB2M; these issues include the dissemination of accurate and timely information, as well as access to testing, care, and services that address the broad range of psychosocial issues that impact GB2M.

## Abbreviations

AOR: adjusted odds ratio
ASO: AIDS service organization
CBO: community-based organization
EI: Employment Insurance
GB2M: gay, bisexual, and two-spirited men, as well as other men who have sex with men (sexual minority men)
LGBT: lesbian, gay, bisexual, and transgender
MISM: men who use the Internet to seek sex with other men
ODSP: Ontario Disability Support Program
OR: odds ratio
PEP: postexposure prophylaxis
PHA: people living with HIV/AIDS
STI: sexually transmitted infection
UAI: unprotected anal intercourse
